# Research Capacity and Culture of Podiatrists in Canada (Quebec) and France: A Parallel Cross‐Sectional Study

**DOI:** 10.1002/jfa2.70227

**Published:** 2026-10-14

**Authors:** Solène Libier, Sergio Cortez Ghio, Antoine Perrier, Magali Brousseau‐Foley, Peter Lazzarini, Virginie Blanchette

**Affiliations:** ^1^ Department of Human Kinetics Université du Québec à Trois‐Rivières Trois‐Rivières Québec Canada; ^2^ VITAM—Research Centre on Sustainable Health Québec Québec Canada; ^3^ In Silico Data Science, Research Support Department Québec Quebec Canada; ^4^ Podiatry Unit, Diabetes Department APHP Pitie Salpêtrière Hospital Paris France; ^5^ Centre intégré universitaire de santé et de services sociaux de la Mauricie et du Centre‐du‐Québec (CIUSSS‐MCQ) Affiliated to Université de Montréal, Faculty of Medicine University Family Medicine Group Trois‐Rivières QC Canada; ^6^ School of Public Health and Social Work Queensland University of Technology Brisbane QLD Australia; ^7^ Allied Health Research Collaborative The Prince Charles Hospital Metro North Hospital and Health Service Brisbane QLD Australia

**Keywords:** capacity building, healthcare services research, podiatry, research

## Abstract

**Introduction:**

Research capacity and culture (RCC) are essential for high‐quality care. However, RCC remains poorly documented among French‐speaking podiatrists. This study aimed to assess RCC among podiatrists in Quebec, Canada, and France at the individual, team, and organizational levels, and to compare RCC scores between both groups.

**Method:**

A parallel, cross‐sectional study was conducted targeting all licensed podiatrists practicing in Quebec, Canada (*n* = 304) and France (*n* = 14,208) in 2023. The data collection was carried online using a validated French version of the RCC tool items at the individual, team, and organizational levels using an 11‐point scale ranging from 0 (no ability) to 10 (perfect ability). Mean RCC item scores (±SEM) were compared between Quebec and France respondents, with exploratory item‐level analyses. Regression analyses were also used to determine associations between sociodemographic characteristics and overall RCC scores.

**Results:**

A total of 2272 podiatrists initiated the survey (127 from Quebec and 2145 from France). Overall, mean individual RCC scores were low, but significantly higher in respondents from Quebec than those from France (3.32 vs. 2.75; *p* < 0.05). Team scores (2.29 vs. 1.82; *p* < 0.05) and organizational scores were also low overall, but significantly higher in respondents from Quebec than those from France (2.31 vs. 1.84; *p* < 0.05). Higher overall RCC scores were significantly associated with more dedicated research time, active research involvement, and research training; among Quebec podiatrists, practice outside private practice was also associated with higher RCC scores (all, *p* < 0.05).

**Conclusion:**

This is the first analysis of RCC among French‐speaking podiatrists, and overall RCC levels were low. Higher RCC scores were associated with active research involvement, dedicated research time, research training, and, among Quebec podiatrists, practice outside private practice. These findings highlight potential targets for capacity‐building initiatives to strengthen evidence‐based podiatry in French‐speaking contexts.

## 1 Introduction

To optimize healthcare outcomes, it is recommended that healthcare professionals demonstrate sufficient research capacity and culture (RCC) to effectively integrate evidence‐based practice into clinical care [1, 2]. Evidence‐based practice combines clinical expertise, patient preferences, and research evidence [1, 3]. This enables informed, appropriate, and effective care by improving knowledge and skills, leading to patient‐centered and efficiently managed practices [2, 4]. Thus, RCC plays a key role in supporting the maturation of healthcare professions and the delivery of optimal healthcare, including in podiatry [5]. Podiatry is a healthcare profession that focuses on the assessment and management of foot and ankle disorders [6]. Although it has been more historically established in English‐speaking contexts, it has gained increased recognition in French‐speaking regions in recent years, notably under the title of *podiatre* (Quebec, Canada) and *pédicure‐podologue* (France). However, the two professions differ in their training pathways, scope of practice, professional autonomy, and legal frameworks. Notably, podiatrists in Quebec complete a 4‐year university‐based Doctorate in Podiatric Medicine, whereas French podiatrists complete a 3‐year State Diploma. Further details are provided in Supporting Information S1: Appendix 1 [7].These developments, particularly the growing emphasis on evidence‐based care, have contributed to improved clinical integration and patient outcomes in these contexts [6, 8].

RCC are recognized as core competencies for the scholarly healthcare professionals, underpinning the ability to develop, appraise, and apply evidence‐based practice, and are embedded in several academic curricula including those in podiatry [9, 10, 11]. Research capacity is defined as the power or ability to conduct or understand research [12], whereas research culture refers to the research environment, including how the team or organization values research, reflected in factors such as incentives, leadership, resources, infrastructure, and available technologies [13]. Therefore, limited RCC may reduce healthcare professionals' capacity to generate, appraise, and apply high‐quality evidence, which may in turn hinder evidence‐based clinical practice and ultimately affect patient health and well‐being.

Despite substantial growth in foot and ankle research over recent decades, podiatry remains an emerging research field, with scientific output representing a small proportion of global research production and being concentrated in a limited number of countries [14, 15]. For this progress to accelerate, there must be sufficient capacity to conduct research effectively [16, 17, 18]. Despite a strong reported interest in research among podiatrists, overall low levels of RCC have been reported among podiatrists in Australia and the United Kingdom, consistent with low levels of research capacity reported across other allied health professions [19, 20, 21]. However, to our knowledge, there have been no studies investigating levels of RCC among French‐speaking podiatrists. This lack of information may, in part, be due to the French language itself representing an additional barrier to research and knowledge dissemination, notably because most scientific publications and databases are predominantly in English [22]. Therefore, this project aimed to evaluate RCC among podiatrists in Quebec, Canada and France at the individual, team, and organizational levels. In addition, we compared mean RCC scores between both groups and explored item‐specific differences and associations between sociodemographic characteristics and RCC.

## 2 Material & Method

### 2.1 Study Design and Ethical Consideration

We conducted a parallel, cross‐sectional survey among podiatrists practicing in French‐speaking Quebec and France. The study followed the CHERRIES (Checklist for Reporting Results of Internet E‐Surveys) [23]. The Institutional Human Research Ethics Committee approved this study (UQTR‐CER‐23‐304‐07.13). All participants provided informed consent at the beginning of the survey.

### 2.2 Population, Recruitment and Sample Size

Eligible participants included all licensed podiatrists and registered with the professional organizations in Quebec (*n* = 304) and France (*n* = 14,208) in 2023. Inclusion criteria were: in Quebec, podiatrists holding a Doctorate in Podiatric Medicine (DPM) and in France, podiatrists holding the national undergraduate degree in podiatry (Diplôme d’État, DE). All participants were required to be able to understand, read, and write in French. The survey was distributed via e‐mail through professional organizations (i.e., *Ordre des podiatres du Québec, Association des podiatres du Québec, and Ordre des pédicures‐podologues*), with reminders sent every 2 weeks. Additional recruitment methods included posts in community groups on social media (e.g., Facebook, LinkedIn) and a two‐minute recruitment video before a research presentation session at an annual Quebec podiatry conference. The survey ran from March to July 2024.

The minimum required sample size was estimated using a widely applied power calculation for psychometric validation studies, namely at least 10 participants per item [24, 25]. As the RCC tool comprises 55 items, a minimum sample size of 550 participants was targeted for the overall cohort; the substantially larger French sample ensured that this requirement was met, while analytical strategies robust to unequal group sizes were applied to account for the imbalance between groups.

### 2.3 Data Collection

The online survey consisted of three sections: demographic characteristic items, RCC items, and three open‐ended question items designed to explore research needs, barriers and facilitators. The data collected in the first two sections are the focus of this manuscript, as the later findings are reported elsewhere in French (Libier, March 2026; in press Le podologue).

Demographic characteristic items included biological sex, gender, age group, location of qualification, years of professional experience, time spent in practice, as well as practice setting (i.e., private practice, hospital, private or university clinic), type of practice (i.e., alone or with other healthcare professionals of the same or other professions), mode of practice (i.e., full‐time or part‐time, permanent or temporary), information on research involvement, training courses attended, and time allocated to research activities. Research involvement was assessed as a separate categorical variable and was not derived from RCC scores. The RCC items had previously been translated, adapted, and validated in French by our team [26, 27], demonstrating satisfactory reliability and validity for assessing RCC at the individual, team, and organizational levels. This comprised 55 self‐reported items scored on an 11‐point scale ranging from 0 (no ability) to 10 (perfect ability) (Supporting Information S1: Appendix 2). Notably, unlike previous studies [21, 28], we introduced an “I don't know” option as a possible response to each item to prevent respondents from arbitrarily selecting extreme values (such as 1 or 10) or entering random values in case of uncertainty, thus reducing measurement bias. This modification was also validated as part of our previous work [27].

The online survey was available via Qualtrics (Qualtrics LLC, USA) through a QR code or link. Qualtrics' optional duplicate detection feature, based on device and browser metadata, was enabled to prevent duplicate entries, in accordance with recommendations for improving the quality of web‐based surveys [23]. Participants had to complete each section before proceeding to the next. Skip logic was implemented to ensure that participants were directed to questions relevant to their profession context (i.e., Quebec podiatrists in Quebec or French podiatrists in France). Participants could withdraw from the survey at any time by closing the browser, and any unanswered questions were treated as missing data.

### 2.4 Analysis

All statistical analyses were performed using R (v4.2.0) in RStudio (v2022.02.3) [29, 30]. Descriptive statistics were performed for categorical variables (N, %) and continuous variables (mean, standard deviation) using the *summary tools* package (v1.0.1) [31]. To maximize sample size and safeguard against bias, and in the absence of consensus on construct assembly for the RCC questionnaire, mean RCC scores were calculated separately at the individual, team, and organizational levels, and an overall RCC score was computed as the mean across all items. Scores were calculated only when a participant had non‐missing responses for all items within the corresponding level. Participants with missing items in each level were excluded from analyses involving that level but could still contribute to other levels for which their responses were complete. As the RCC tool has no validated cut‐offs, the use of terms such as “low” refers only to the relative position of mean scores on the 0–10 scale.

Overall RCC mean scores as well as mean scores at the individual, team, and organization levels were compared between Quebec and French podiatrists with analyses of variance of aligned rank transformed data using the *ARTool* package (v0.11.1) [32]. This non‐parametric approach was favored because model residuals in the parametric framework violated assumptions of normality and homogeneity of variance, as assessed with the *performance* package (v0.15.1) [33]. To explore item‐level differences between Quebec and French podiatrists, non‐parametric repeated‐measure analyses of variance were conducted using the *nparLD* package (v2.2) [34] to account for the within‐subject correlation across items. Post hoc non‐parametric permutation tests were then conducted, with *p*‐values adjusted for multiple comparisons using the Benjamini–Hochberg method. Finally, to explore the association between demographic characteristics and overall RCC mean score, we fitted random forest regression models separately for Quebec and French podiatrists using the *randomForest* package (v4.7‐1.1) [35]. Predictor importance was then ranked by the permutation‐based mean decrease in out‐of‐bag accuracy. To better understand the potential association of top predictors with the overall mean RCC score, univariate analyses of variance of aligned rank transformed data were then run for each of them. At this stage, variable level recoding was conducted when necessary and informed by data visualizations. Some multi‐category variables were collapsed into fewer categories prior to regression analyses to avoid sparse cells and ensure model stability. Post hoc Tukey tests were run using the *emmeans* package (v1.8.6) when needed [36]. All demographic variables that were collected were screened through this process. For all analyses, group means, standard errors of the mean (SEM)s, and *p*‐values are reported herein. SEMs were reported rather than SDs to emphasize the precision of group mean estimates, given the bounded nature of RCC scores (0–10).

## 3 Results

### 3.1 Participation Rate and Participants' Characteristics

Out of 304 podiatrists invited from Quebec and 14,208 from France, 127 (42%) and 2145 (15%) initiated the survey, respectively (2272 in total). The demographic characteristics of both groups are shown in Table 1. Participants in both contexts were mostly women. In Quebec, the largest age group was 30–39 years, whereas in France, participants were more evenly distributed across age groups. Most podiatrists in both contexts worked full‐time on a permanent basis in private practice. Compared with their French counterparts, Quebec podiatrists more often reported 6–15 years of experience and working in practices with at least one other podiatrist, whereas French podiatrists more frequently reported more than 16 years of practice and worked alone. Work status (permanent vs. temporary/other) was similar in both contexts (94.1% vs. 96.9%; *p* = 0.11). In contrast, no significant differences were observed for sex (*p* = 0.86), gender (*p* = 0.53), practice setting (*p* = 0.41), and work status (*p* = 0.11), whereas significant differences emerged for age group, years of practice, and type of practice (all *p* < 0.001).

**TABLE 1:** Demographic characteristics.

Variable	Categories	Quebec podiatrists (*N* = 127) Count (valid %)	French podiatrists (*N* = 2145) Count (valid %)	*p*‐value^a^
Sex	Female	88 (69.3)	1505 (70.2)	0.86
	Male	39 (30.7)	636 (29.7)	
	Prefer not to answer	0 (0)	4 (0.2)	
	Missing	0	0	
Gender	Women	88 (69.3)	1494 (69.7)	0.53
	Men	39 (30.7)	631 (29.4)	
	Other/Prefer not to answer	0 (0)	20 (0.9)	
	Missing	0	0	
Age group	20–29 years	34 (28.8)	310 (16.0)	< 0.001
	30–39 years	68 (57.6)	623 (32.2)	
	40–49 years	10 (8.5)	519 (26.9)	
	50–59 years	5 (4.2)	347 (18.0)	
	≥ 60 years	1 (0.8)	133 (6.9)	
	Missing	9	213	
Number of years of practice	Less than 2 years	11 (9.3)	115 (6.0)	< 0.001
	3–5 years	24 (20.3)	178 (9.3)	
	6–10 years	36 (30.5)	338 (17.6)	
	11–15 years	37 (31.4)	332 (17.3)	
	More than 16 years	9 (7.7)	961 (49.9)	
	No longer practicing	1 (0.8)	0 (0)	
	Missing	9	221	
Type of practice	Practice with at least one other person	102 (86.4)	1168 (60.4)	< 0.001
	Practice alone	14 (11.9)	737 (38.1)	
	Other	2 (1.7)	30 (1.6)	
	Missing	9	210	
Practice setting	Private practice	110 (93.2)	1761 (91.0)	0.41
	Other	8 (6.8)	174 (9.0)	
	Missing	9	210	
Work status	Permanent full‐time/part‐time	111 (94.1)	1874 (96.9)	0.11
	Temporary/Other	7 (5.9)	61 (3.2)	
	Missing	9	210	

^a^ *χ* ^2^ or Fisher's exact tests.

### 3.2 Research Profile of the Sample

Research interest and involvement were generally low in both groups. In France, only 8.3% were actively involved in research, while 51.1% selected “No interest in research” as their research involvement category. Among Quebec podiatrists, 11.0% were involved in research and 31.4% expressed interest, whereas only 24.6% reported no interest, which contrasts sharply with the French group (51.1%). This substantial difference in disinterest between the two groups may partly contribute to the lower RCC scores observed among French podiatrists. Few had advanced research training (Master's or Doctoral degrees) (1.7% of Quebec podiatrists; 5.3% of French podiatrists), and while about half of the participants in both contexts reported attending non‐credit courses, only French podiatrists reported credit‐bearing courses (55.9% vs. 0%). Most dedicated less than 10% of their time to research (Table 2). Significant differences were observed for research involvement and credit‐bearing training (both *p* < 0.001), whereas no significant differences were found for non‐credit courses (*p* = 0.17), Master's/Doctoral training (*p* = 0.08), and time dedicated to research (*p* = 0.67) (Table 2).

**TABLE 2:** Questions used to assess the research profile.

Variable	Questions	Quebec podiatrists (*N* = 127) Count (valid %)	French podiatrists (*N* = 2145) Count (valid %)	*p*‐value^a^
Involvement in research	Involved in research	13 (11.0)	160 (8.3)	< 0.001
	Limited to scholarly activities during professional training.	39 (33.1)	406 (21.0)	
	Interested in research but have not yet had the opportunity to participate in research activities.	37 (31.4)	380 (19.6)	
	No interest in research.	29 (24.6)	989 (51.1)	
	Missing	9	210	
Research training	Non‐credit courses (i.e. not counted toward mandatory continuing education) (Yes)	61 (51.7)	1125 (59.5)	0.17
	Credit courses (i.e. counted toward mandatory continuing education) (Yes)	0 (0)	1081 (55.9)	< 0.001
	Completed or uncompleted Master's/Doctoral training (Yes)	2 (1.7)	103 (5.3)	0.08
	Missing	9	210	
Percentage of time spent on research	≥ 10%	14 (11.9)	183 (9.5)	0.67
	< 10%	40 (33.9)	655 (33.9)	
	No time	64 (54.2)	1097 (56.7)	
	Missing	9	210	

^a^ *χ* ^2^ or Fisher's exact tests.

### 3.3 Comparison of RCC Mean Scores Between French and Quebec Podiatrists

We detected significant differences in the RCC overall mean score and across the three levels between groups. Quebec podiatrists consistently reported higher mean scores than their French counterparts (all *p* < 0.001). These results are presented in Table 3.

**TABLE 3:** Scores for each level of research capacity and culture.

Levels	Quebec podiatrists		French podiatrists		*p* value^a^
	*N*	(Mean ± SEM)	N	(Mean ± SEM)	
Individual	104	3.32 ± 0.17	1169	2.75 ± 0.05	< 0.001
Team	79	2.29 ± 0.21	839	1.82 ± 0.05	< 0.001
Organizational	70	2.31 ± 0.22	662	1.84 ± 0.07	< 0.001
Overall	67	2.50 ± 0.20	543	2.03 ± 0.06	< 0.001

Abbreviation: SEM, Standard Error of the Mean.

^a^ Analysis of variance of aligned rank transformed data.

### 3.4 Item‐Specific Comparisons Between French and Quebec Podiatrists

At each of the three levels, Quebec podiatrists scored significantly higher mean scores than French podiatrists in several items (Tables 4, 5, 6). At the individual level, Quebec podiatrists reported higher mean scores for finding relevant literature, critically reviewing literature, using referencing systems, submitting ethics applications, and integrating research findings into practice. At the team level, Quebec podiatrists reported higher mean scores for research planning, access to resources, mentoring, and access to literature and software. At the organizational level, Quebec podiatrists reported higher mean scores for managerial support, evidence‐guided planning, and promotion of evidence‐based practice. Items significant at *p* < 0.05 are presented in Tables 4, 5, 6, while pairwise comparisons for all items are provided in Supporting Information S1: Appendix 2.

**TABLE 4:** Item‐specific RCC scores that differed between French and Quebec podiatrists at the individual level.

Items^a^	Quebec podiatrists (mean ± SEM)	French podiatrists (mean ± SEM)	Adjusted *p* value
Individual level	*N* = 104	*N* = 1169	
Q1 finds relevant literature	7.43 ± 0.22	5.33 ± 0.08	< 0.001
Q2 critically reviews the literature	6.39 ± 0.20	4.45 ± 0.08	< 0.001
Q3 uses a computer referencing system (e.g. Endnote)	3.24 ± 0.28	2.59 ± 0.08	0.004
Q6 submits an ethics application	2.15 ± 0.23	1.60 ± 0.05	0.021
Q14 integrates research findings into practice	5.51 ± 0.29	3.56 ± 0.09	< 0.001

Abbreviations: Q, Question or item; SEM, Standard error of the mean.

^a^ All items are presented in Supporting Information S1: Appendix 2.

**TABLE 5:** Item‐specific RCC scores that differed between French and Quebec podiatrists at the team level.

Items^a^	Quebec Podiatrists (Mean ± SEM)	French podiatrists (Mean ± SEM)	Adjusted *p* value
Team level	*N* = 79	*N* = 839	
Q1 provides resources to support staff research training	3.17 ± 0.28	2.35 ± 0.08	< 0.001
Q2 provides funds, equipment or administration to support research activities	1.86 ± 0.18	1.58 ± 0.05	0.006
Q3 does team level planning for research development	2.13 ± 0.24	1.76 ± 0.06	0.009
Q5 has team leaders that support research	2.29 ± 0.26	1.87 ± 0.07	0.009
Q6 provides opportunities to get involved in research	2.53 ± 0.27	1.90 ± 0.07	< 0.001
Q7 does planning that is guided by evidence	3.48 ± 0.32	1.77 ± 0.06	< 0.001
Q10 provides access to literature searching and article retrieval	3.09 ± 0.36	1.89 ± 0.07	< 0.001
Q12 supports applications for research scholarships or degrees	1.84 ± 0.24	1.44 ± 0.05	0.024
Q17 has incentives and support for mentoring activities	2.19 ± 0.27	1.60 ± 0.06	0.004
Q19 supports peer‐reviewed publication of research	2.54 ± 0.32	1.81 ± 0.07	0.006
Q20 provides software to support research activities	1.86 ± 0.24	1.41 ± 0.05	0.022

Abbreviations: Q, Question or item; SEM, Standard error of the mean.

^a^ All items are presented in Supporting Information S1: Appendix 2.

**TABLE 6:** Item‐specific RCC scores that differed between French and Quebec podiatrists at the organisational level.

Items^a^	Quebec Podiatrists (Mean ± SEM)	French podiatrists (Mean ± SEM)	Adjusted *p* value
Organisational level	N = 70	N = 662	
Q1 provides resources to support staff research training	2.16 ± 0.27	1.83 ± 0.08	0.048
Q4 provides access to literature search and article retrieval	2.69 ± 0.32	1.87 ± 0.08	0.008
Q5 has senior managers that support research	2.37 ± 0.29	1.84 ± 0.08	0.023
Q7 ensures organization planning is guided by evidence	3.34 ± 0.37	1.76 ± 0.08	< 0.001
Q10 promotes clinical practice based on evidence	4.81 ± 0.42	2.35 ± 0.10	< 0.001
Q11 encourages research activities relevant to practice	3.54 ± 0.37	2.56 ± 0.10	< 0.001
Q18 supports applications for research scholarships or degrees	2.09 ± 0.29	1.53 ± 0.07	0.023
Q19 supports the peer‐reviewed publication of research	2.60 ± 0.35	1.74 ± 0.08	0.008

Abbreviations: Q, Question or item; SEM, Standard error of the mean.

^a^ All items are presented in Supporting Information S1: Appendix 3. As this is all the data for all items. And Table 4 highlights only the significant results.

### 3.5 Predictors of Overall RCC Scores

Random Forest regression models were then used to explore potential predictors of the overall RCC mean score among demographic variables, as presented in Tables 7 and 8. Research involvement and time allocated to research emerged as the strongest predictors in both groups. The smaller sample sizes reflect the presence of missing data for some demographic variables. Univariate analyses of variance of aligned rank transformed data confirmed significant associations with overall RCC scores (all *p* < 0.001). In France, postgraduate training (Master's/PhD) was associated with higher scores (*p* < 0.01). In Quebec, where postgraduate training is uncommon, practice setting was an additional factor associated with RCC (*p* = 0.001).

**TABLE 7:** Demographic variables associated with overall RCC scores among Quebec podiatrists.

Predictor variable	Category/Level	*N*	Mean ± SEM	*p*‐value
Involvement in research	Involved in research	7	5.55 ± 0.76	< 0.001
	Little or no involvement in research	60	2.15 ± 0.15	
Percentage of time spent on research	≥ 10%	8	5.17 ± 0.83	< 0.001
	< 10%	20	2.80 ± 0.32	
	No time	39	1.80 ± 0.12	
Practice setting	Private practice	63	2.25 ± 0.16	< 0.001
	Other	4	6.56 ± 0.68	

*Note:* Only categories significantly associated with RCC scores are presented. Non‐significant categories (e.g., certificate‐level research training) were tested but are not shown.

Abbreviation: SEM, Standard Error of the Mean.

**TABLE 8:** Demographic variables associated with overall RCC scores among French podiatrists.

Variable	Level	N	Mean ± SEM	*p*‐value^a^
Involvement in research	Involved in research	49	4.49 ± 0.30	< 0.001
	Little or no involvement	494	1.79 ± 0.05	
Percentage of time spent on research	≥ 10%	56	3.74 ± 0.32	< 0.001
	< 10%	188	2.27 ± 0.11	
	No time	299	1.56 ± 0.05	
Research training: Master or doctoral training completed	No	505	1.87 ± 0.06	< 0.001
	Yes	38	4.18 ± 0.41	
Years of practice	Less than 25 years	435	2.13 ± 0.08	< 0.001
	More than 26 years	108	1.64 ± 0.11	

Abbreviation: SEM, Standard Error of the Mean.

^a^ Analysis of variance of aligned rank transformed data.

## 4 Discussion

This study assessed and compared RCC among podiatrists in Quebec and France, across individual, team, and organizational levels, and explored the influence of sociodemographic factors. Overall, mean RCC scores were low in absolute terms on the 0–10 scale, although Quebec podiatrists consistently reported significantly higher mean scores than French podiatrists across individual, team, organizational, and overall levels. In both groups, research involvement and allocated time were the strongest factors, suggesting that active engagement matters more than background. For French podiatrists, postgraduate training (Master's/Doctoral) was associated with higher RCC scores, whereas in Quebec practice setting, it was an additional factor associated with RCC. These findings are consistent with those from rural Australian health services, where staffing levels, workload, and available resources influenced how research was supported and prioritized [37].

First, higher education and research training have also been associated with greater research involvement among other health professionals, including nurses, allied health professionals, and dietitians [38, 39].

Second, age and years of experience may partly reflect differences in exposure to research training. In France, the integration of podiatry into the university continuum in 2012 strengthened the place of evidence‐based practice and research in initial training [40], whereas many participants were likely trained before these reforms. In Quebec, most participants were trained at UQTR, where basic research training is mandatory [11], which may partly contribute to the higher RCC scores observed.

Third, practice setting was associated with RCC scores among Quebec podiatrists. Those practicing outside private practice had higher overall RCC scores than those working in private practice. However, this finding should be interpreted cautiously because the “Other” category included only four participants and grouped different practice settings. More broadly, previous research has shown that contextual factors such as workforce constraints, infrastructure, and institutional support can influence research engagement and capacity [37].

More broadly, previous studies suggest that research engagement among podiatrists is influenced by access to institutional resources and collaborative research environments [20]. This may be particularly relevant in our sample, as most participants worked in private practice, where access to research infrastructure and collaboration opportunities may be more limited [7, 41]. Some sociodemographic and professional characteristics, including years of practice, practice setting, postgraduate education, and research involvement, may also have influenced participants' responses and contributed to differences between groups.

### 4.1 Overall Level of Research Capacity and Culture

Overall, average RCC scores were low in both contexts, although Quebec podiatrists scored significantly higher than French podiatrists. These differences remained limited and did not reflect a generalized higher research capacity. The overall RCC score we reported should be interpreted cautiously, as it represents an exploratory adaptation rather than a validated construct of the RCC tool. These findings are consistent with previous studies showing that allied health professionals tend to have lower RCC levels than medical professionals [42], a trend also reported among other podiatrists [20, 21, 28], dietitians [43], speech‐language pathologists [44], occupational therapists [45], social workers [46], and other paramedical professionals [26, 47].

However, several RCC items, displayed in Supporting Information S1: Appendix 2, showed significant differences in favor of Quebec podiatrists. At the individual level, items Q1,Q2 and Q14 displayed the most pronounced differences, with higher scores among Quebec podiatrists. These items reflect fundamental research skills typically introduced during initial training, while lower‐scoring items such as Q5 and Q6 correspond to more advanced competencies often acquired through postgraduate education. These differences may reflect variations in the structure or content of initial training programs or differing professional orientations.

These findings align with earlier research using the same RCC tool across other health professions, which similarly show confidence in fundamental skills but a lack of experience with more complex stages of the research process [20, 28]. However, unlike in our study, a previous study involving allied health professionals reported higher scores at the team and organizational levels than at the individual level [48]. This difference may reflect contextual factors, such as greater institutional support or more collaborative research structures in certain healthcare systems. Methodological differences between our study and previous RCC studies, including our choice to aggregate items using the mean, may explain some variation.

At the team level, most items revealed differences between professions, especially Q7 and Q10, where Quebec podiatrists again scored higher. These items reflect a more structured, evidence‐based approach to clinical practice, likely supported by better access to research resources and a more established research culture. The high score on Q7 among Quebec podiatrists suggests stronger integration of evidence into practice, possibly influenced by training or organizational support. For both groups, however, Q9 and Q12 received consistently low scores, indicating limited team and financial support that may hinder the development of research capacity. As reported in previous studies with Australian podiatrists and dietitians, low scores in these areas often reflect a lack of organizational and financial support for research, which limits its integration into clinical practice [21, 43].

Interestingly, item Q1 received one of the highest scores in both groups, highlighting a shared recognition of the importance of training healthcare professionals in research. This may reflect a broader cultural shift toward valuing research competencies and integrating them into clinical practice [49]. For French podiatrists, item Q8 stands out as one of the few where they scored higher than their Quebec counterparts, suggesting that patient involvement in research may be more strongly emphasized or valued in this group.

At the organizational level, items Q7 and Q10 again show notable differences between the two groups. These items suggest that Quebec podiatrists may work in environments where clinical research and evidence‐based practice are more supported, possibly due to organizational priorities or a stronger culture of continuous improvement. For both groups, however, the lowest scores on Q3 and Q9 suggest that organizational planning and financial support for research remain limited despite recognition of its importance. Comparable limitations have been reported across other allied health contexts, where insufficient planning, financial support, and institutional leadership hinder the development of research skills and capacity [37, 50].

Conversely, high scores on items Q10 and Q11 suggest that research and evidence‐based practice are valued at the organizational level. However, overall organizational‐level scores remained among the lowest for both groups. Some studies have reported higher organizational scores than those observed in our sample, but these were mostly conducted in hospital settings, where structures are more formalized and research capacity is more fully integrated into day‐to‐day operations [21, 48]. This contrast likely reflects the more research‐oriented and institutionally supportive nature of hospital environments than private practice [20].

### 4.2 Strengths and Limitations

First, the use of the French version of the RCC tool makes comparisons with studies using the original RCC tool more difficult. Moreover, the length of the French version and the complexity of some items may have led to respondent fatigue, potentially reducing completion rates [51]. Secondly, although we have reached the minimum sample size required for meaningful analysis [24], we may not fully represent all the diversity of professionals in each group, which limits the generalizability of the results. However, the detailed description of the sample and measures may support the transferability of the findings to similar contexts [52]. Participants who are more inclined toward research or have higher skills or an interest in research are more likely to have responded to our survey. Thus, there is a potential selection bias that could lead to an overestimation of RCC levels among the most engaged podiatrists. However, as the overall rate is relatively low, this bias would not necessarily change the fact that our results demonstrate the need to strengthen the RCC of podiatrists.

A strength of this study lies in the exploratory item‐level analyses, which highlighted the specific capacity most responsible for between‐group differences, offering more detailed insights than aggregated scores alone. However, while we introduced an overall RCC score to provide a synthetic overview, this measure has not been validated and should be considered exploratory. Similarly, analyses based on Random Forest feature importance should be interpreted with caution, as they are exploratory. As no validated cut‐offs exist for the RCC tool, the interpretation of overall scores relies solely on the relative position of mean values on the 0‐10 scale. In addition, given the large sample size, some statistically significant differences may reflect small effect sizes, and *p*‐values should therefore be interpreted in relation to the magnitude of group differences. However, the two groups also shared similar characteristics, and strong statistical methods were used to support valid comparisons.

Finally, this was the first study to provide an overview of RCC in French‐speaking podiatric contexts; it represents an important first step toward better understanding the situation and supporting future capacity‐building efforts within and across contexts. Further qualitative studies would help to deepen the interpretation of these results and better understand the underlying mechanisms. In that perspective, the present results allowed us to generate hypotheses to guide future research.

### 4.3 Practical Implications and Further Research

Our results can help to build RCC in podiatry as we highlighted areas requiring additional support, training, and access to resources at different levels. Several studies have shown that RCC can be improved at different levels [53], but focusing solely on individuals or policy alone is insufficient to drive sustainable change [54]. In addition to the individual level, we added knowledge regarding team and organization levels to support RCC. This can promote the transition to high‐quality, evidence‐based podiatric medicine and enable decision‐makers responsible for education or legislative bodies to make more informed decisions.

Nevertheless, at the individual level, capacity can be developed through optimal training and mentoring based on RCC tool capacity [55], or by pursuing research‐oriented graduate studies. At the team level, having dedicated research time, training opportunities, and peer support can strengthen the collective capacity [53]. Podiatrists can also contribute meaningfully to interdisciplinary clinical research teams, particularly when integrated into hospital‐based or academic research structures, which helps foster collaboration, research culture, and knowledge translation [20, 56]. This can also have leverage effects on the organizational level, systems and structures [57, 58]. For example, combining clinical work with research roles can boost engagement, even though this is easier to put in place in public settings/systems [28, 59]. Comparable approaches have also been developed in the United Kingdom, where clinical academic career pathways and research internship programmes for nurses and allied health professionals, including podiatrists, have combined research training, mentorship, and opportunities to remain engaged in clinical practice while developing research skills [60, 61]. In Australia, one study followed podiatrists over a year during which research capacity‐building strategies were introduced. Their initial results were similar to ours, but scores improved significantly after the intervention, especially at the organizational level [53]. This clearly demonstrates how access to support and resources can influence RCC, something that our results highlighted in the French‐speaking podiatry context.

## 5 Conclusion

This study is the first to assess RCC among French‐speaking podiatrists using a validated and culturally adapted tool. Our results showed that the overall RCC levels remain low among podiatrists in both Quebec and France. Quebec podiatrists consistently reported significantly higher scores than their French counterparts at the overall, individual, team, and organizational levels. Item‐level analyses confirmed that all significant differences were higher among Quebec podiatrists. By providing new knowledge in an understudied context, this study lays the groundwork for future efforts aimed at strengthening RCC in French‐speaking podiatry and collaboration. These efforts could focus on improving initial and continuing education, as well as creating more conducive environments for podiatric research, as podiatrists, whatever their language, should be important contributors to the medical research field on the foot and ankle.

## Author Contributions

**Solène Libier:** conceptualization, methodology, investigation, data curation, formal analysis, writing – original draft, writing – review and editing. **Sergio Cortez Ghio:** conceptualization, methodology, formal analysis, writing – review and editing. **Antoine Perrier:** conceptualization, formal analysis, writing – review and editing. **Magali Brousseau‐Foley:** conceptualization, validation, writing – review and editing. **Peter Lazzarini:** conceptualization, validation, writing – review and editing. **Virginie Blanchette:** conceptualization, methodology, formal analysis, funding acquisition, supervision, writing the original draft, writing – review and editing, project administration.

## Funding

This project was supported by the Ordre des Podiatres du Québec and Réseau‐1 Québec.

## Conflicts of Interest

The authors declare no conflicts of interest.

## Supporting information


Supporting Information S1

## Data Availability

The data that supports the findings of this study are available in the supplementary material of this article.
