# Supplementary material for: Research Capacity and Culture of Podiatrists in Canada (Quebec) and France: A Parallel Cross‐Sectional Study
Source: J Foot Ankle Res. 2026 Oct 14;19(4):e70227. doi: 10.1002/jfa2.70227 (PMC13669538; doi:10.1002/jfa2.70227)
Supplement: Supplementary file 1 [file JFA2-19-e70227-s001.docx]

**Appendix 1. Comparison of Quebec Podiatrist and French Podiatrist**

One key distinction is that the minimum entry-level qualification differs between Québec and France, with podiatrists in Quebec completing a doctoral degree and those in France holding a state diploma. Although both professions fall under the same clinical specialty (foot health), they differ in scope of practice, level of professional autonomy and legal framework. The table below demonstrates a few notable differences. However, please refer to the scope of practice via the Order websites, given the rapid evolution of these professions [6, 40].

| **Comparison Items** | **Quebec Podiatrist** | **French Podiatrist** |
| --- | --- | --- |
| Specialty | Foot health | Foot health |
| Training | Undergraduate doctorate (4 years – Podiatric Medicine) that lead to a DPM delivered by the University | State Diploma (3 years post-high school) that lead to a DE (diplôme d’état) delivered by a private school.  Some schools are affiliated to the University. |
| Professional status | Regulated medical profession | Regulated allied health profession |
| Legal framework and Order | Ordre des podiatres du Québec  Law on podiatry | Ordre national des pédicures-podologues |
| Medical diagnosis | Yes, with prescriptions | Yes, but limited and without prescriptions |
| Prescriptions | Topical, oral medications including local intradermal, intra-articular injections, imagery (X-rays, ultrasound), dressing | Topical treatments, dressings |
| Surgery | Soft tissue minor surgeries  Bone surgery if the podiatrist as the complementary training (surgical residency 1-3 more years) | Not authorized |
| Foot orthotics | Yes – design, prescription, fabrication and adjustment | Yes – design and fabrication |
| Autonomy | High: independent practice, primary care provider, no referrals are needed | Less autonomy: usually following medical referral |
| Overall objective | Comprehensive prevention and care for foot pathologies that are not systemic problems. | Local treatments, posture correction, prevention. |
| Coverage | Not covered by public health insurance (Régie de l’assurance maladie du Québec (RAMQ)) but partially reimbursed by most private insurance plans.  Private practice. | Some consultations and orthotics are partially reimbursed by public health insurance; private insurance is often needed for full coverage.  Private and public practices. |

**Appendix 2. RCC tool items and item-specific scores in French and Quebec podiatrists**

| Questions / Items | Quebec Podiatrists (Mean ± SEM) | French podiatrists (Mean ± SEM) | Adjusted *p* value^†^ |
| --- | --- | --- | --- |
| Individual level | n = 104 | n = 1169 |  |
| Q1 Finds relevant literature | 7.43 ± 0.22 | 5.33 ± 0.08 | **< 0.001** |
| Q2 Critically reviews the literature | 6.39 ± 0.20 | 4.45 ± 0.08 | **< 0.001** |
| Q3 Uses a computer referencing system (e.g. Endnote) | 3.24 ± 0.28 | 2.59 ± 0.08 | **0.004** |
| Q4 Writes a research protocol | 2.40 ± 0.22 | 2.42 ± 0.07 | 0.515 |
| Q5 Secures research funding | 1.74 ± 0.18 | 1.44 ± 0.04 | 0.159 |
| Q6 Submits an ethics application | 2.15 ± 0.23 | 1.60 ± 0.05 | **0.018** |
| Q7 Designs questionnaires | 2.47 ± 0.21 | 2.59 ± 0.07 | 0.653 |
| Q8 Collects data, e.g. surveys, interviews | 3.13 ± 0.27 | 2.85 ± 0.08 | 0.242 |
| Q9 Uses computer data management systems | 2.86 ± 0.25 | 2.63 ± 0.07 | 0.215 |
| Q10 Analyses qualitative research data | 2.44 ± 0.20 | 2.59 ± 0.07 | 0.774 |
| Q11 Analyses quantitative research data | 2.86 ± 0.24 | 2.63 ± 0.07 | 0.242 |
| Q12 Writes a research report | 2.73 ± 0.23 | 2.44 ± 0.07 | 0.120 |
| Q13 Writes for publication in peer-reviewed journal | 2.28 ± 0.21 | 2.08 ± 0.06 | 0.072 |
| Q14 Integrates research findings into practice | 5.51 ± 0.29 | 3.56 ± 0.09 | **< 0.001** |
| Q15 Provides advice to less experienced researchers | 2.12 ± 0.19 | 2.10 ± 0.06 | 0.343 |
| Team level | n = 79 | n = 839 |  |
| Q1 Provides resources to support staff research training | 3.18 ± 0.28 | 2.35 ± 0.08 | **< 0.001** |
| Q2 Provides funds, equipment or administration to support research activities | 1.86 ± 0.18 | 1.58 ± 0.05 | **0.003** |
| Q3 Does team level planning for research development | 2.13 ± 0.24 | 1.76 ± 0.06 | **0.009** |
| Q4 Ensures staff involvement in developing that plan | 2.06 ± 0.22 | 1.84 ± 0.06 | 0.103 |
| Q5 Has team leaders that support research | 2.29 ± 0.26 | 1.87 ± 0.07 | **0.009** |
| Q6 Provides opportunities to get involved in research | 2.53 ± 0.27 | 1.90 ± 0.07 | **0.003** |
| Q7 Does planning that is guided by evidence | 3.48 ± 0.32 | 1.77 ± 0.06 | **< 0.001** |
| Q8 Has consumer involvement in research activities or planning | 2.24 ± 0.24 | 2.32 ± 0.08 | 0.280 |
| Q9 Has applied for external funding for research | 1.63 ± 0.21 | 1.47 ± 0.05 | 0.518 |
| Q10 Provides access to literature searching and article retrieval | 3.09 ± 0.36 | 1.89 ± 0.07 | **< 0.001** |
| Q11 Conducts research activities relevant to practice | 2.42 ± 0.29 | 2.17 ± 0.08 | 0.330 |
| Q12 Supports applications for research scholarships or degrees | 1.84 ± 0.24 | 1.44 ± 0.05 | **0.025** |
| Q13 Has mechanisms to monitor research quality | 1.78 ± 0.20 | 1.65 ± 0.06 | 0.066 |
| Q14 Provides experts accessible for research advice | 1.97 ± 0.25 | 1.76 ± 0.07 | 0.105 |
| Q15 Disseminates research results at research forums or seminars | 2.03 ± 0.26 | 1.78 ± 0.07 | 0.271 |
| Q16 Supports a multi-disciplinary approach to research | 2.52 ± 0.29 | 2.29 ± 0.08 | 0.130 |
| Q17 Has incentives and support for mentoring activities | 2.19 ± 0.27 | 1.60 ± 0.06 | **< 0.001** |
| Q18 Has external partners (e.g. universities) engaged in research | 2.14 ± 0.29 | 1.75 ± 0.07 | 0.137 |
| Q19 Supports peer-reviewed publication of research | 2.54 ± 0.32 | 1.81 ± 0.07 | **0.005** |
| Q20 Provides software to support research activities | 1.86 ± 0.24 | 1.41 ± 0.05 | **0.020** |
| Organizational level | n = 70 | n = 662 |  |
| Q1 Provides resources to support staff research training | 2.16 ± 0.26 | 1.83 ± 0.08 | **0.040** |
| Q2 Provides funds, equipment or admin to support research activities | 1.63 ± 0.18 | 1.58 ± 0.07 | 0.392 |
| Q3 Has a plan or policy for research development | 1.56 ± 0.19 | 1.68 ± 0.07 | 0.838 |
| Q4 Provides access to literature search and article retrieval | 2.69 ± 0.32 | 1.87 ± 0.08 | **0.010** |
| Q5 Has senior managers that support research | 2.37 ± 0.29 | 1.84 ± 0.08 | **0.020** |
| Q6 Ensures staff career pathways are available in research | 1.74 ± 0.20 | 1.68 ± 0.07 | 0.071 |
| Q7 Ensures organization planning is guided by evidence | 3.34 ± 0.37 | 1.76 ± 0.08 | **< 0.001** |
| Q8 Has consumers involved in research | 2.11 ± 0.26 | 2.08 ± 0.09 | 0.308 |
| Q9 Accesses external funding for research | 1.5 ± 0.16 | 1.45 ± 0.06 | 0.264 |
| Q10 Promotes clinical practice based on evidence | 4.81 ± 0.42 | 2.35 ± 0.10 | **< 0.001** |
| Q11 Encourages research activities relevant to practice | 3.54 ± 0.37 | 2.56 ± 0.10 | **0.006** |
| Q12 Provides software programs for analysing research data | 1.73 ± 0.23 | 1.56 ± 0.06 | 0.444 |
| Q13 Has mechanisms to monitor research quality | 1.81 ± 0.24 | 1.75 ± 0.07 | 0.607 |
| Q14 Provides experts for research advice | 1.86 ± 0.26 | 1.69 ± 0.07 | 0.444 |
| Q15 Supports a multi-disciplinary approach to research | 2.36 ± 0.29 | 2.31 ± 0.10 | 0.444 |
| Q16 Provides forums or bulletins to present research findings | 1.86 ± 0.25 | 1.71 ± 0.07 | 0.444 |
| Q17 Engages external partners (e.g. universities) in research | 1.96 ± 0.27 | 1.80 ± 0.08 | 0.392 |
| Q18 Supports applications for research scholarships or degrees | 2.09 ± 0.28 | 1.53 ± 0.07 | **0.025** |
| Q19 Supports the peer-reviewed publication of research | 2.6 ± 0.34 | 1.74 ± 0.08 | **0.012** |
| Q20 Requires ethics approval for research activities | 2.41 ± 0.34 | 1.97 ± 0.09 | 0.285 |

Abbreviation: Q =Question or item; SEM: Standard error or the mean

^†^Repeated measures analysis of variance. Permutation-based post hoc tests and Benjamini–Hochberg adjustment.

**Bold** characters indicates that the difference between groups is significant at α = 0.05.

**Appendix 3. RCC tool items and item-specific scores in French and Quebec podiatrists, with number of respondents per item**

| Level | Items | n respondents Quebec | n respondents France | p-value | p-value adjusted |
| --- | --- | --- | --- | --- | --- |
| **Individual** | Q1 Finds relevant literature* | 114 | 1460 | < 0.001 | < 0.001 |
|  | Q2 Critically reviews the literature* | 114 | 1460 | < 0.001 | < 0.001 |
|  | Q3 Uses a computer referencing system (e.g. Endnote)* | 114 | 1460 | 0.001 | 0.004 |
|  | Q4 Writes a research protocol | 114 | 1460 | 0.435 | 0.501 |
|  | Q5 Secures research funding | 114 | 1460 | 0.085 | 0.159 |
|  | Q6 Submits an ethics application* | 114 | 1460 | 0.007 | 0.021 |
|  | Q7 Designs questionnaires | 114 | 1460 | 0.627 | 0.671 |
|  | Q8 Collects data, e.g. surveys, interviews | 114 | 1460 | 0.164 | 0.240 |
|  | Q9 Uses computer data management systems | 114 | 1460 | 0.134 | 0.223 |
|  | Q10 Analyses qualitative research data | 114 | 1460 | 0.790 | 0.790 |
|  | Q11 Analyses quantitative research data | 114 | 1460 | 0.176 | 0.240 |
|  | Q12 Writes a research report | 114 | 1460 | 0.061 | 0.130 |
|  | Q13 Writes for publication in peer-reviewed journal | 114 | 1460 | 0.028 | 0.700 |
|  | Q14 Integrates research findings into practice* | 114 | 1460 | < 0.001 | < 0.001 |
|  | Q15 Provides advice to less experienced researchers | 114 | 1460 | 0.267 | 0.334 |
| **Team** | Q1 Provides resources to support staff research training* | 105 | 1232 | < 0.001 | < 0.001 |
|  | Q2 Provides funds, equipment or administration to support research activities* | 105 | 1232 | 0.001 | 0.003 |
|  | Q3 Does team level planning for research development* | 105 | 1232 | 0.004 | 0.009 |
|  | Q4 Ensures staff involvement in developing that plan | 105 | 1232 | 0.068 | 0.097 |
|  | Q5 Has team leaders that support research* | 105 | 1232 | 0.004 | 0.008 |
|  | Q6 Provides opportunities to get involved in research* | 105 | 1232 | 0.001 | 0.003 |
|  | Q7 Does planning that is guided by evidence* | 105 | 1232 | < 0.001 | < 0.001 |
|  | Q8 Has consumer involvement in research activities or planning | 105 | 1232 | 0.250 | 0.277 |
|  | Q9 Has applied for external funding for research | 105 | 1232 | 0.523 | 0.523 |
|  | Q10 Provides access to literature searching and article retrieval* | 105 | 1232 | < 0.001 | < 0.001 |
|  | Q11 Conducts research activities relevant to practice | 100 | 1087 | 0.302 | 0.318 |
|  | Q12 Supports applications for research scholarships or degrees* | 100 | 1087 | 0.014 | 0.025 |
|  | Q13 Has mechanisms to monitor research quality | 100 | 1087 | 0.043 | 0.071 |
|  | Q14 Provides experts accessible for research advice | 100 | 1087 | 0.063 | 0.097 |
|  | Q15 Disseminates research results at research forums or seminars | 100 | 1087 | 0.235 | 0.276 |
|  | Q16 Supports a multi-disciplinary approach to research | 100 | 1087 | 0.092 | 0.123 |
|  | Q17 Has incentives and support for mentoring activities* | 100 | 1087 | 0.001 | 0.003 |
|  | Q18 Has external partners (e.g. universities) engaged in research | 100 | 1087 | 0.110 | 0.137 |
|  | Q19 Supports peer-reviewed publication of research* | 100 | 1087 | 0.003 | 0.008 |
|  | Q20 Provides software to support research activities* | 100 | 1087 | 0.011 | 0.022 |
| **Organisation** | Q1 Provides resources to support staff research training* | 97 | 954 | 0.017 | 0.042 |
|  | Q2 Provides funds, equipment or admin to support research activities | 97 | 954 | 0.256 | 0.393 |
|  | Q3 Has a plan or policy for research development | 97 | 954 | 0.839 | 0.839 |
|  | Q4 Provides access to literature search and article retrieval* | 97 | 954 | < 0.001 | < 0.001 |
|  | Q5 Has senior managers that support research* | 97 | 954 | 0.005 | 0.016 |
|  | Q6 Ensures staff career pathways are available in research | 97 | 954 | 0.034 | 0.075 |
|  | Q7 Ensures organization planning is guided by evidence* | 97 | 954 | < 0.001 | < 0.001 |
|  | Q8 Has consumers involved in research | 97 | 954 | 0.197 | 0.328 |
|  | Q9 Accesses external funding for research | 97 | 954 | 0.135 | 0.270 |
|  | Q10 Promotes clinical practice based on evidence* | 97 | 954 | < 0.001 | < 0.001 |
|  | Q11 Encourages research activities relevant to practice* | 93 | 877 | < 0.001 | < 0.001 |
|  | Q12 Provides software programs for analysing research data | 93 | 877 | 0.403 | 0.448 |
|  | Q13 Has mechanisms to monitor research quality | 93 | 877 | 0.578 | 0.608 |
|  | Q14 Provides experts for research advice | 93 | 877 | 0.396 | 0.448 |
|  | Q15 Supports a multi-disciplinary approach to research | 93 | 877 | 0.398 | 0.448 |
|  | Q16 Provides forums or bulletins to present research findings | 93 | 877 | 0.366 | 0.448 |
|  | Q17 Engages external partners (e.g. universities) in research | 93 | 877 | 0.290 | 0.414 |
|  | Q18 Supports applications for research scholarships or degrees* | 93 | 877 | 0.009 | 0.026 |
|  | Q19 Supports the peer-reviewed publication of research* | 93 | 877 | 0.003 | 0.012 |
|  | Q20 Requires ethics approval for research activities | 93 | 877 | 0.172 | 0.313 |

Abbreviation: Q refers to Question or items.

*Statistically significant difference.
